# Useful old casts: a comment on Hansford & Turvey (2018), ‘Unexpected diversity within the extinct elephant birds (Aves: Aepyornithidae)’

**DOI:** 10.1098/rsos.181826

**Published:** 2019-02-06

**Authors:** Eric Buffetaut, Cédric Audibert, Jérôme Tabouelle, Delphine Angst

**Affiliations:** 1 CNRS (UMR 8538), Laboratoire de Géologie, Ecole Normale Supérieure, PSL Research University, 24 rue Lhomond, 75231 Paris Cedex 05, France; 2 Musée des Confluences, Centre de conservation et d'étude des collections, 13A rue Bancel, 69007 Lyon, France; 3 Fabrique des Savoirs, Réunion des Musées Métropolitains, 7 cours Gambetta, 76500 Elbeuf, France; 4 School of Earth Sciences, University of Bristol, Life Sciences Building, 24 Tyndall Avenue, Bristol BS8 1TQ, UK

In their valuable revision of aepyornithiform systematics, Hansford & Turvey [1] deplore the fact that some significant specimens could not be measured and included in their morphometric analysis, because they have been destroyed or cannot currently be located. The most important specimens in this regard probably are the skeletal elements in the type series of *Aepyornis maximus* Geoffroy Saint-Hilaire, 1851 [2], the first aepyornithiform taxon to have been described, which could not be located in the collections of the Muséum National d'Histoire Naturelle in Paris, where they should be. Hansford and Turvey have designated as the lectotype of *Aepyornis maximus* an incomplete left tarsometatarsus, lacking the proximal end and a large part of the shaft, but showing the three distal trochleae. This is a wise choice, since that specimen was the best preserved in the original collection studied (but not illustrated) by Geoffroy Saint-Hilaire [2] and was used by him to establish the fact that the huge eggs with which it was (loosely) associated were indeed those of a giant bird. As noted by Owen [3], the set of bones available to Geoffroy Saint-Hilaire also included an incomplete right tarsometatarsal and the proximal end of a right fibula. Because the original specimen of the lectotype was not available, Hansford and Turvey did not include it in their analysis, although they did mention a few measurements published by Owen [3]—others were given by Geoffroy Saint-Hilaire [2]. Additional measurements could have been obtained from the natural size lithographs of that specimen, based on a cast, published by Bianconi [4]. However, we suggest that a number of measurements of the newly designated lectotype can be obtained from one of the many casts of the specimen that were distributed throughout Europe and beyond soon after Geoffroy Saint-Hilaire's original description [5]. In the course of an ongoing project on *Aepyornis* specimens in French collections, we have located a number of them in various museums. Of special importance in this respect is a set of casts (collective collection number 050303038) kept in the collections of the Natural History Museum in Rouen, in Normandy (figure 1). It consists of high-quality and well-preserved plaster casts of the three bones originally examined by Geoffroy Saint-Hilaire in 1851, *viz.* two incomplete tarsometatarsi (including the lectotype designated by Hansford and Turvey) and the proximal end of a fibula [3], which can be considered as plastosyntypes (following Evenhuis's nomenclature [6]). The records of the Rouen Museum indicate that the specimens (plus a cast of an egg), sent by the National Museum of Natural History in Paris, were received in March 1852, a little more than a year after Geoffroy Saint-Hilaire's presentation of the originals at the French Academy of Sciences. There is therefore no doubt that these are casts of the syntype series of *Aepyornis maximus* (no other *Aepyornis* specimens were available in Paris at that time). This is confirmed by a comparison with the measurements provided by Geoffroy Saint-Hilaire [1] and Owen [3] and with the lithograph published by Bianconi [4]. The cast of the lectotype (or plastolectotype [6], figure 2) shows that it lacked the proximal end and part of the shaft and that trochleae II and III were incompletely preserved. However, many of the measurements listed by Hansford and Turvey for the tarsometatarsus (24 out of 44) can be taken on it. Although the casts in Rouen are of excellent quality, various other casts of the lectotype of *Aepyornis maximus* are kept in other museums and could serve the same purpose. For instance, Lydekker [7], in his *Catalogue of the fossil birds in the British Museum (Natural History)* [today the Natural History Museum], lists under collection number A 81 a cast of an incomplete tarsometatarsus figured by Bianconi [4] that is clearly a cast of the lectotype of *Aepyornis maximus*. The nineteenth century habit of widely distributing casts of important palaeontological specimens can still have beneficial consequences today when originals have been destroyed or lost.

**Figure 1.**
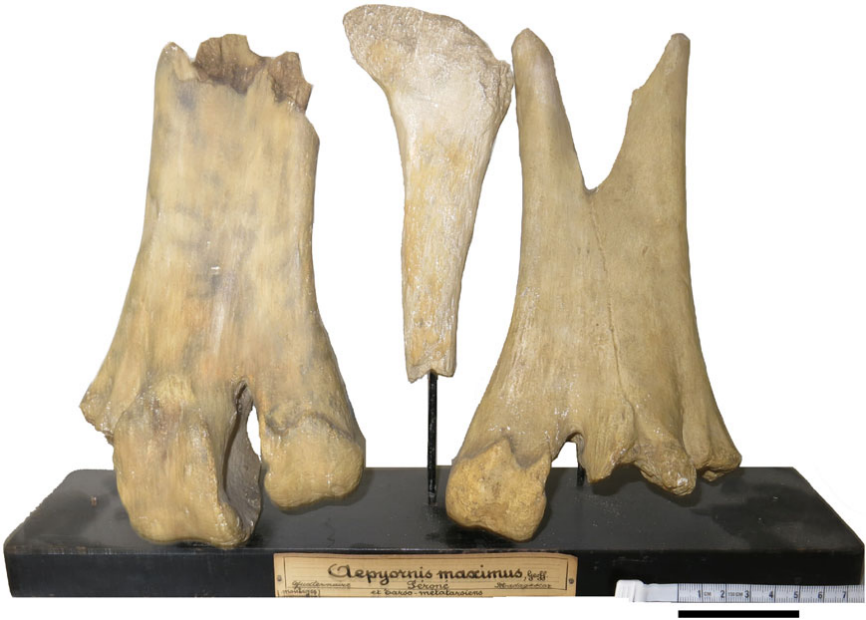
Set of casts of the bones of *Aepyornis maximus* mentioned by Geoffroy Saint-Hilaire [2] (plastosyntype series), Natural History Museum, Rouen, collective number 050303038. From left to right: incomplete left tarsometatarsus (plastolectotype), proximal end of right fibula, incomplete right tarsometatarsus. Scale bar: 5 cm.

**Figure 2.**
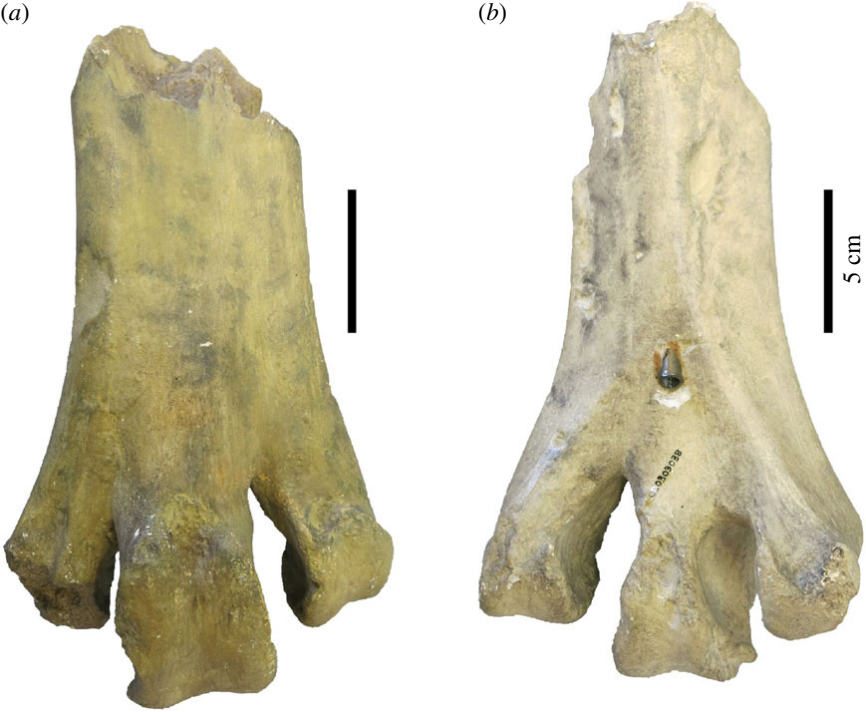
Plastolectotype, i.e. cast of the lectotype (as designated by Hansford and Turvey [1]) of *Aepyornis maximus*, an incomplete left tarsometatarsus, Natural History Museum, Rouen. (*a*) Dorsal view and (*b*) plantar view. The small brass tube inserted into the plantar face of the bone makes it possible to insert it on a brass wire for display in a vertical position (figure 1).

## Data accessibility

This article has no additional data.

## References

[1] Hansford JP, Turvey ST 2018 Unexpected diversity within the extinct elephant birds (Aves: Aepyornithidae) and a new identity for the world's largest bird. R. Soc. open sci. 5, 181295 (10.1098/rsos.181295) 30839722 PMC6170582

[2] Geoffroy Saint-Hilaire I 1851 Note sur des ossements et des œufs trouvés à Madagascar, dans des alluvions modernes, et provenant d'un Oiseau gigantesque. C. R. Acad. Sc. Paris 32, 101–107.

[3] Owen R 1852 Note on the eggs and young of the *Apteryx*, and on the casts of the eggs and certain bones of the *Aepyornis* (Isid. Geoffroy), recently transmitted to the Zoological Society of London. Proc. Zool. Soc. London 19-20, 9–13.

[4] Bianconi G 1865 Dell'*Aepyornis maximus* e del tarso-metatarso degli uccelli. Mem. Accad. Sc. Ist. Bologna 5, 63–140.

[5] Milne-Edwards A, Grandidier A 1869 Nouvelles observations sur les caractères zoologiques et les affinités naturelles de l'*Aepyornis* de Madagascar. Ann. Sc. Nat. 12, 167–196.

[6] Evenhuis NL 2008 A compendium of zoological type nomenclature: a reference source. Bishop Mus. Tech. Rep. 41, 1–23.

[7] Lydekker R 1891 Catalogue of the fossil birds in the British Museum (Natural History). London, UK: Trustees of the British Museum (Natural History).

